# Deep generative learning of location-invariant visual word recognition

**DOI:** 10.3389/fpsyg.2013.00635

**Published:** 2013-09-19

**Authors:** Maria Grazia Di Bono, Marco Zorzi

**Affiliations:** ^1^Computational Cognitive Neuroscience Lab, Department of General Psychology, University of PadovaPadova, Italy; ^2^IRCCS San Camillo Neurorehabilitation HospitalVenice-Lido, Italy

**Keywords:** orthographic coding, open-bigrams, connectionist modeling, hierarchical generative models, deep unsupervised learning

## Abstract

It is widely believed that orthographic processing implies an approximate, flexible coding of letter position, as shown by relative-position and transposition priming effects in visual word recognition. These findings have inspired alternative proposals about the representation of letter position, ranging from noisy coding across the ordinal positions to relative position coding based on open bigrams. This debate can be cast within the broader problem of learning location-invariant representations of written words, that is, a coding scheme abstracting the identity and position of letters (and combinations of letters) from their eye-centered (i.e., retinal) locations. We asked whether location-invariance would emerge from deep unsupervised learning on letter strings and what type of intermediate coding would emerge in the resulting hierarchical generative model. We trained a deep network with three hidden layers on an artificial dataset of letter strings presented at five possible retinal locations. Though word-level information (i.e., word identity) was never provided to the network during training, linear decoding from the activity of the deepest hidden layer yielded near-perfect accuracy in location-invariant word recognition. Conversely, decoding from lower layers yielded a large number of transposition errors. Analyses of emergent internal representations showed that word selectivity and location invariance increased as a function of layer depth. Word-tuning and location-invariance were found at the level of single neurons, but there was no evidence for bigram coding. Finally, the distributed internal representation of words at the deepest layer showed higher similarity to the representation elicited by the two exterior letters than by other combinations of two contiguous letters, in agreement with the hypothesis that word edges have special status. These results reveal that the efficient coding of written words—which was the model's learning objective—is largely based on letter-level information.

## Introduction

Visual word recognition and reading aloud is one of the cognitive domains where connectionist modeling has achieved its greatest success. Following seminal studies published in the 1980s (McClelland and Rumelhart, 1981; Rumelhart and McClelland, 1982; Seidenberg and McClelland, 1989), recent modeling work has produced highly detailed simulations of skilled reading, reading development, and dyslexia (e.g., Plaut et al., 1996; Zorzi et al., 1998; Harm and Seidenberg, 1999; Coltheart et al., 2001; Perry et al., 2007, 2010, 2013; Zorzi, 2010; Ziegler et al., in press; see Zorzi, 2005, for a review). Nevertheless, despite an impressive up-scaling of connectionist models of reading in recent years (e.g., Perry et al., 2010, 2013), most of these models remain largely underspecified with regard to the “visual front-end” of the reading system. That is, most models stipulate that the identity and position of individual letters is coded in a way that is abstracted from the retinal input both in terms of shape and spatial location with respect to eye fixation. In particular, the latter assumption implies a location-invariant word-centered representation, with letters aligned according to a fixed template (e.g., left-justified slot-based coding). The issue of how location-invariance might be computed from the native retinotopic (eye-centered) code has recently attracted much interest (Dehaene et al., 2005; Dandurand et al., 2010; Hannagan et al., 2011), because it is closely tied to a lively debate on the nature of orthographic coding and more specifically on the coding of letter position during visual word recognition (e.g., Whitney, 2001; Grainger and van Heuven, 2003; Davis and Bowers, 2006; Gomez et al., 2008; Davis, 2010; Grainger and Ziegler, 2011).

The theoretical debate on letter position coding was triggered by studies that reported relative-position and transposition priming effects in visual word recognition using the masked priming paradigm (e.g., Humphreys et al., 1990; Peressotti and Grainger, 1999; Perea and Lupker, 2003; Schoonbaert and Grainger, 2004; Grainger et al., 2006). The first phenomenon refers to the finding that word recognition is facilitated when primes are composed of a subset of letters constituting the target word, but only when relative positions are respected. Transposition priming, instead, refers to the finding that when primes share all the constituent letters of the target words, priming still persists when small changes in letter order is performed (e.g., transposing two adjacent letters). It is widely believed that these priming effects stem from a level of orthographic processing where some form of approximate, flexible coding of letter positions operates (Grainger, 2008) and have inspired alternative models of letter position coding (Grainger and Whitney, 2004; Gomez et al., 2008; Davis, 2010). All models share the assumption that visual word recognition is built upon parallel processing of the constituent letters, in contrast to an holistic word-shape representation (see Pelli et al., 2003; Grainger, 2008; Grainger et al., 2012). From the computational point of view, holistic word-shape coding is extremely costly because it requires to solve shape invariance for each word rather than for each letter of the alphabet. However, the models differ in terms of how approximate letter position coding is achieved. For example, the Overlap model of Gomez et al. (2008) assumes a noisy coding of letter position within the classic slot-based coding scheme used in the interactive-activation model (IAM) of McClelland and Rumelhart (1981). In the IAM model, words are processed in parallel from a set of letter detectors that are length-dependent and position-specific. Uncertainty about letter positions is implemented in the Overlap model as a Gaussian distribution of activation across the ordinal positions in the word. Letter position uncertainty is also a central feature of Davis' (2010) spatial coding model.

A different theoretical perspective is that orthographic coding is based on combinations of contiguous and non-contiguous ordered letter pairs, in a way to code relative rather than absolute letter positions (Whitney, 2001; Grainger and van Heuven, 2003). For instance, the word WITH would be coded with the set of bigrams [WI, WT, WH, IT, IH, TH], a scheme known as Open-bigram coding (Grainger and Whitney, 2004). Open-bigrams are an intermediate coding between the representation of single letters and whole-words. Grainger and van Heuven (2003) propose the existence of a bank of letter detectors performing parallel letter identification, independently from the physical characteristics of the letters (i.e., shape and size) but not from the spatial location. Therefore, the activity of letter detectors is an abstract representation of letters conveying information about letter identity at a specific locations. In the next stage, a more abstract “relative position map” is formed, coding for the relative position of letter identities within the word, independently from their shape and size, and independently from the spatial location of the word (i.e., location invariance). According to the open-bigram model, this is possible through a bank of open-bigram units, receiving the input from the letter detectors: the open bigram for a specific ordered letter pair (e.g., A_C) is activated by all the possible location combinations in the letter detectors for the given letter order. Open-bigrams then send their activations to all compatible word representations. In this way a flexible relative-position code mediates the processing of reading words as a whole.

The idea that visual word recognition might involve a number of intermediate and progressively more abstract levels of orthographic coding is the key aspect of Dehaene et al.'s (2005) local combination detector (LCD) model. Though not implemented as a computer simulation, the LCD model is inspired by the neurophysiology of the primate visual object recognition system. Specifically, object recognition is based on hierarchical processing of basic local features that are gradually integrated into more complex and abstract features (through increasing size of receptive fields) to progressively achieve invariance for size, shape, and location (see Riesenhuber and Poggio, 1999, for a computational model). Given that reading is a recent cultural invention, it is unlikely that the brain contains a specific neural mechanism for visual word recognition. Thus, learning to recognize printed words independently from their location, font, size, etc. might be achieved by recycling the cortical machinery for object recognition (Dehaene and Cohen, 2011). According to the LCD model, part of the occipito-temporal “what” pathway is organized into a hierarchy of neuronal levels, each composed of local combination detectors that are gradually sensitive, through increasing complexity and size of their receptive fields, to larger fragments of words. Besides the well-known finding that the occipito-temporal cortex of skilled readers contains a “visual word form area” (Cohen et al., 2002; Cohen and Dehaene, 2004), recent functional neuroimaging studies support the LCD model by showing that perception of written words involves the sensitivity to increasingly larger visual units along a posterior-to-anterior gradient in the ventral visual stream (Vinckier et al., 2007). Notably, open bigrams are important intermediate-size units in the LCD model.

The problem of learning a location-invariant orthographic representation of printed words was recently tackled by Dandurand et al. (2010) with connectionist simulations. They used error backpropagation to train a feedforward neural network with one layer of hidden units on the mapping from location-specific letter identities to location-invariant localist word representations. The phenomena of transposed-letter and relative-position priming were investigated in the network by presenting stimuli obtained by transposing two letters or removing one letter from a trained target word. The transposed letter stimuli, compared to control stimuli in which the two letters were replaced by non-constituent ones, produced an activation pattern that was more similar to that produced by the target word. In the same vein, stronger similarity to the target word activation was obtained when the input stimuli maintained the letter order (e.g., ABC for ABCD) with respect to controls in which the letter order was reversed (e.g., CBA for ABCD). Moreover, when the order was maintained, stimuli composed of non-contiguous letters yielded a stronger similarity to the target word in comparison to stimuli containing only the contiguous letters (e.g., ABD vs. ABC for ABCD). These findings suggested that the network had learned a code for contiguous and non-contiguous letter combinations. Hannagan et al. (2011) further investigated the neural network model of Dandurand et al. (2010) by analyzing its hidden layer activity. They found that no knowledge about bigrams was learned by the network. Instead, the network learned letter identities almost independently from their locations (in a “semi-location-invariant” way). This information allowed to compute constituent bigrams and words without the explicit coding of letter combinations. These results are in line with the overlap model of Gomez et al. (2008).

While the connectionist studies of Dandurand et al. (2010) and Hannagan et al. (2011) represent a first important attempt to understand how a location-specific letter-based code could be mapped onto location invariant word representations, the plausibility of the model is hindered by its network architecture and by the use of supervised learning by error backpropagation. Besides the well-known lack of biological plausibility of the backpropagation algorithm (O'Reilly, 1998), the supervised learning regimen is problematic because it implies that orthographic learning requires an external, explicit teaching signal at each word encounter. Moreover, the classic feedforward network with one layer of hidden units used by Dandurand and colleagues does not capture the hierarchical organization of the visual system, which is a key feature for achieving invariant object recognition in biologically inspired computational models of vision (Riesenhuber and Poggio, 1999).

In this article we present a connectionist model of location-invariant visual word recognition that can be cast within the broader theoretical framework of Dehaene et al.'s (2005) LCD model. The assumption that orthographic learning exploits the cortical machinery for object recognition leads to the prediction that perceptual invariance for visual words might emerge from unsupervised generative learning in a neural network with a hierarchical architecture, that is a “Deep Belief Network” (DBN; Hinton, 2007; Stoianov and Zorzi, 2012; Zorzi et al., 2013). DBNs are stochastic recurrent neural networks with many layers of hidden units that encode increasingly more complex features of the sensory input across layers (Hinton and Salakhutdinov, 2006; Hinton, 2007, 2013). In practice, a DBN is a stack of Restricted Boltzmann Machines (RBMs; Hinton, 2002) trained in a layer-wise fashion. RBMs are stochastic networks with one layer of visible neurons encoding the input patterns and one layer of hidden neurons connected through bidirectional symmetric links. Learning in RBMs is unsupervised and its objective is to build internal representations of the sensory input by fitting a generative model to the data. Therefore, after training all RBM layers in succession, the DBN is a hierarchical generative model in which the latent causes of the data are represented through distributed non-linear representations across hidden layers (HLs). DBNs represent the state-of-the-art in machine learning but they are also particularly appealing for connectionist modeling of cognition because they learn multiple levels of representation without any supervision or reward and they have a sound probabilistic formulation (see Zorzi et al., 2013, for a tutorial review). Crucially, the analyses of the internal representations can reveal an emergent coding strategy that closely mirrors single-cell recording data (e.g., Lee et al., 2008; De Filippo De Grazia et al., 2012a; Stoianov and Zorzi, 2012). In the present work we trained a DBN on an artificial dataset of letter strings presented at five possible retinal locations. We asked whether location-invariant word recognition would emerge from unsupervised deep learning and what type of intermediate coding would support the transition between location-specific (i.e., eye-centered) letter coding and location-invariant word representations.

## Materials and methods

We employed a DBN with three HLs for learning a generative model of written words. In the following subsections, we describe the training dataset and the network architecture. The code (Matlab/Octave) used for the simulation and the training set is available at http://ccnl.psy.unipd.it/deeplearning.

### Training dataset

We used an artificial dataset constructed *ad hoc* in order to investigate orthographic learning in a restricted but tightly controlled way (also see Dandurand et al., 2010). The dataset was composed of 120 3-letter words presented at 5 different eye-centered locations (one central and two locations on each side of the central one), for a total of 600 (120 words × 5 locations) input patterns. An artificial lexicon was generated by considering all simple permutations of three letters without repetitions from a partial alphabet composed of six letters (i.e., A B C D E F). In this way, it was possible to balance the frequency of each letter in the lexicon and to avoid letter repetition. Indeed, including letter repetition within the same word could introduce a possible confound in identifying the contribution of open bigrams to the orthographic coding for visual word recognition.

### Input coding

**Figure 1:**
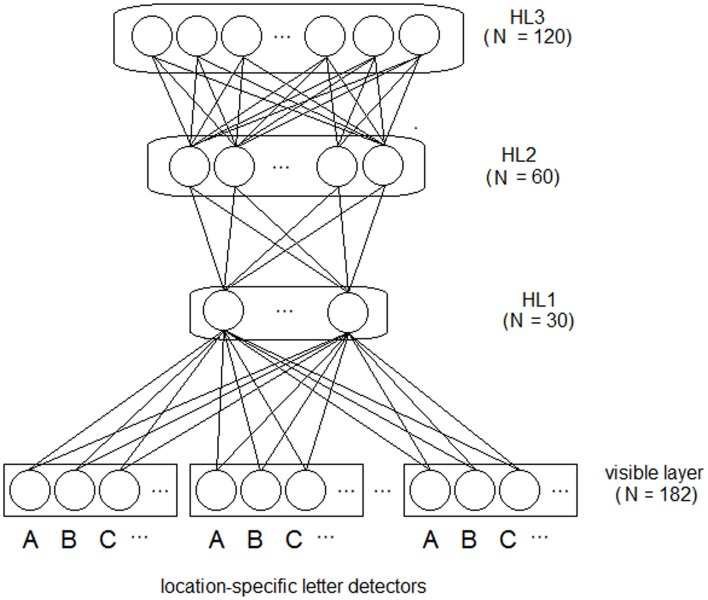
**Architecture of the deep network model.** Letter strings are presented to the visible (i.e., input) layer using a bank of location-specific letter detectors within slots encoding 7 different spatial locations. Activity of the visible layer is fed to three hierarchically organized layers of feature detectors (hidden neurons). All connections are bidirectional and symmetric. Note that word-level information is not explicitly represented in the network and it is not supplied during training.

We used a sparse coding (i.e., slot coding) for representing the training words (see also Dandurand et al., 2010). Input words were coded by the pattern of activity over 7 location-specific (i.e., eye-centered) letter slots (see Figure 1) and each word could be coded at 5 different locations. Each letter within a word was coded by the activation of a specific letter unit (over a set of 26 units, one for each letter of the alphabet^1^), at a specific eye-centered location. Blank locations were coded using zeros for all units of a slot. Thus, the input pattern was a vector of 182 binary values.

### Network architecture

The deep network had one visible layer encoding the input data and three hierarchically organized HLs (see Figure 1). A characteristic of deep networks is that adding HLs generally increases the complexity of the features that can be detected during learning. There is a point, however, where adding further layers does not improve global performance (see Hinton, 2012 for a practical guide). We measured the global performance of the network by linearly decoding training words from the activity of the deepest layer (see Zorzi et al., 2013, for discussion). Thus, we empirically determined the number of layers, starting from a first hidden layer of 30 neurons (approximately corresponding to the square root of the total number of training patterns), then we increased the number of layers, doubling the number of hidden neurons with respect to the previous layer (i.e., 60 and 120 neurons for layer 2 and 3, respectively). Performance did not improve with more than three HLs.

Learning proceeded layer-wise (i.e., one layer at a time) for computational efficiency. For the first hidden layer, the input was the activity of the visible layer. For the other layers, the input was the activity of the previously trained layer. Each RBM (one for each layer), was trained with the Contrastive Divergence (CD) learning algorithm (Hinton and Salakhutdinov, 2006) to learn a generative model of the input data without supervision (i.e., maximizing the likelihood of reconstructing the data). Crucially, no word level information was provided to the network. For each RBM, learning involves two phases: a “positive” and a “negative” phase. During the positive phase the visible units are clamped to the data pattern and their activity (v^+^_i_) spreads to the hidden neurons (h^+^_j_). In the negative phase, a stochastically selected binary state of the hidden neurons (using their state h^+^_j_ as probability to turn them on) feeds back to the visible units (v^−^_i_) through the top-down weights (i.e., reconstruction of the input vector) and then feeds forward again to the hidden neurons (h^−^_j_) (see Zorzi et al., 2013, for a more detailed discussion). The weights w_ij_ are updated with a small learning fraction (η) of the difference between pairwise correlations measured in the positive and negative phases:

$$\Delta W=\eta (v^{+}h^{+}-v^{-}h^{-})$$

We trained the deep network for a maximum number of 1000 epochs, using a learning rate of 0.1, and an increasing momentum ranging between 0.5 and 0.9. To ensure robustness of the results, we trained 10 versions of the same network using different initial random weights.

Unsupervised deep learning was carried out on a multi-core high-performance cluster using an Octave/Open-MPI parallelization (De Filippo De Grazia et al., 2012b; Testolin et al., 2013). Note that Testolin et al. (2013) provide code for a variety of parallel solutions and show that learning time can be further reduced by exploiting the GPUs of low-cost graphic cards on a desktop PC.

## Results

### Decoding from activity of hidden layers

After training, we investigated the quality of the representation generated within each HL. We used a linear classifier for decoding the input words from each of the three HLs; the classifier weights were computed using the pseudo-inverse method (Hertz et al., 1991), which is equivalent to using the delta rule but more efficient and parameter-free (see Zorzi et al., 2013). Only at this level of analysis we introduced word-level information for learning a linear association between the activity of each hidden layer, computed presenting an input word on the visible layer, and the same word used as target. Each target word was coded into a binary output unit, independently from the location at which it was presented. The presence of a corresponding word was marked by a value of 1, its absence by a value of 0. There were 120 output units, each corresponding to a training word, independently from its location. For instance, with 4 target words (e.g., ABC, ABD, ABE, ABF) the input word ##ABC## (as well as ####ABC) would be coded as 1 0 0 0, whereas the word ###ABE# (as well as ABE####) would be coded as 0 0 1 0. Recognition performance was expressed in terms of the percentage of input words correctly decoded, independently from the location.

**Figure 2:**
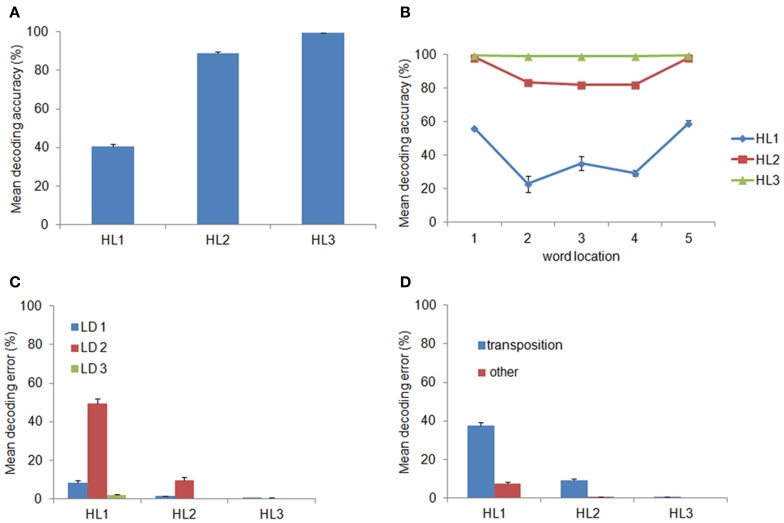
**Word decoding from the Hidden Layers (HLs). (A)** Mean decoding accuracy, expressed in terms of the percentage of correctly recognized words, as a function of layer depth. Decoding accuracy significantly increased across layers and was near-perfect at the deepest layer (i.e., HL3). **(B)** Decoding accuracy as a function of word location. Decoding accuracy in HL1 and HL2 varied among locations, with higher accuracy at the two outer locations, whereas accuracy in HL3 was near-perfect across all locations. **(C)** Decoding error as a function of the Levenshtein Distance (LD) from the correct word and layer depth. Most of the decoding errors were not close neighbors but were at a distance of 2, which include transposition errors. **(D)** Error distribution for *LD* = 2, after splitting it between transposition and other errors, as a function of layer depth. Transposition errors were predominant and they accounted for virtually all errors in HL2 and HL3. Error bars in all graphs indicate standard error across ten simulations using networks with different initial random weights.

We hypothesized that decoding accuracy would increase across layers, thereby indexing that internal representations become more abstract with the increasing of the network's depth. The percentage of correctly decoded words is shown in panel **(A)** of Figure 2 as a function of the layer used as input to the classifier. Indeed, decoding accuracy significantly increased with layer depth [*F*_(1.19, 10.73)_ = 1872.01, *p* < 0.0001, η^2^_*p*_ = 0.99, Greenhouse-Geisser corrected for sphericity] and it reached near-perfect accuracy (*M* = 99.43 ± 0.14 s.e.m.) at the deepest hidden layer (HL3). Panel **(B)** of Figure 2 shows decoding accuracy as a function of location of the input words. Notably, location-invariance increased as a function of layer depth: that is, decoding accuracy in HL1 and HL2 varied among locations (with a tendency for higher accuracy at the two outer locations), whereas accuracy in HL3 was near-perfect across all locations.

**Figure 3:**
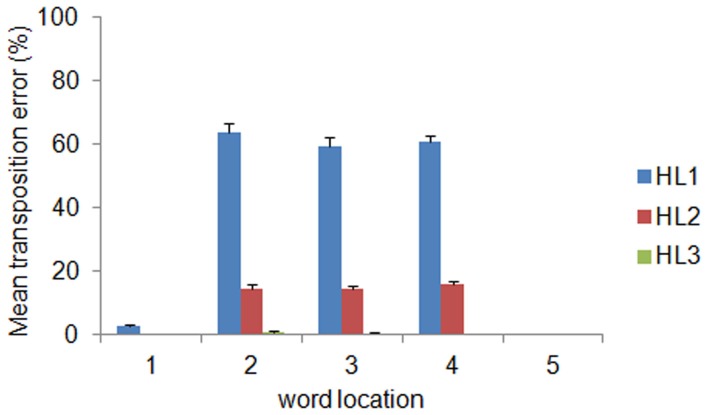
**Mean transposition errors as a function of layer depth and word location.** For HL1 and HL2, transposition errors were mainly and almost similarly distributed across the three inner locations. Error bars indicate standard error across ten simulations.

The distribution of decoding errors can provide insights about how orthographic information is encoded within the different layers of the deep network. We therefore analyzed the decoding error distribution as a function of the orthographic distance between the input word and the incorrectly decoded word, indexed by the Levenshtein Distance (LD) (Yarkoni et al., 2008). For example, the word ABC has a distance of 1 from the words ^*^BC, A^*^C, AB^*^ (where the symbol ^*^ means a letter that does not belong to it), a distance of 2 from the words with transposed letters (i.e., ACB, BAC, CBA, BCA, and CAB) as well as from the words A^**^, ^*^B^*^, ^**^C, and a distance of 3 from all words containing letters that do not belong to it (e.g., DEF, EFD). Note that the LD measure was computed independently from the location of the input word. The error distribution is shown in panel **(C)** of Figure 2 as function of LD and layer depth. Note that the majority of errors consisted in producing words at a distance of 2. The finding that a large proportion of decoding errors do not involve words at the closest orthographic distance (*LD* = 1) but are concentrated on a distance of 2 suggests that most errors might be in fact transposition errors. Splitting the error distribution for *LD* = 2 between words with transposed letters and other words showed that this was indeed the case (see Figure 2D). Finally, we also assessed whether the distribution of transposition errors varied across locations. The results are shown in Figure 3. For HL1 and HL2, transposition errors were mainly and almost similarly distributed across the three inner locations. This result is complementary to the distribution of decoding accuracy across locations (see Figure 2B), which was higher at the two outer locations. This advantage can be readily explained by the fact that training implies less position uncertainty for letters in the outer slots. For example, during training of the word ABC the only letter presented in slot 1 is A, whereas slot 3 can contain the letters A, B, or C. Thus, letter A in the leftmost (or rightmost) slot provides unambiguous evidence for words starting (or ending) with A, whereas letter A in slot 3 may belong to any word that contains A.

### Analysis on single neurons

To further characterize the information encoded into the trained network, we analyzed the activity of each neuron within each HL. This analysis was performed on a single network (i.e., the first network trained). Borrowing the classic method used in single-cell recording studies (also see Stoianov and Zorzi, 2012; Zorzi et al., 2013), we sought to establish whether and how the activity of each neuron is modulated by two key factors: (i) word selectivity; (ii) location invariance. Finally, a further analysis on single neurons allowed us to assess whether knowledge about bigrams had emerged in the network.

### Word selectivity

We fixed the preferred location of each neuron, choosing the location that maximized its activity across all trained words. We then fixed its preferred word, on the basis of its maximum activity at the preferred location. Finally, we performed a linear regression on its normalized activity in response to the training words (presented at the preferred location) using LD as predictor (3 levels: 0, 1, and 2; note that *LD* = 0 indexes the preferred word). We discarded the words at an orthographic distance *LD* = 3 from the input word, which are those words composed of all letters that did not belong to the input word. After False Discovery Rate (FDR) correction for multiple comparisons, we selected all the neurons for which the regression was significant. No word selectivity was found in HL1. In contrast, word selectivity emerged in 95% of HL2 neurons (FDR *p* = 0.037) and in 97.5% of HL3 neurons (FDR *p* = 0.037). Activity of these neurons was modulated in a monotonically decreasing way by the orthographic distance of the input words from the preferred word.

### Word location invariance

**Figure 4:**
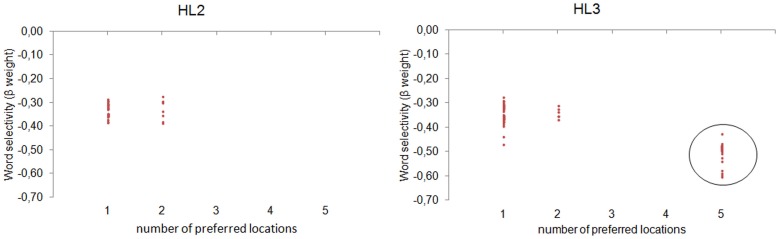
**Analysis on single neurons.** The distribution of neurons in HL2 (left panel) and HL3 (right panel) is shown as a function of the number of preferred locations and the word selectivity index (regression weight of the LD predictor). Only neurons showing a significant modulation of activity in the regression analysis are plotted. Location invariance emerged only in 30.83% of HL3 neurons (circled in black), where word recognition was also near-perfect.

We fixed the preferred word of each neuron, choosing the training word that maximized its activity. Then, we used a pattern matching procedure for assessing the degree of invariance to the spatial location of the preferred word. In particular, we defined a set of binary location vectors, each encoding the preference for one or more specific locations (e.g., 0 0 1 0 0, coding the preference for the central location; 1 1 1 1 1, coding an equal preference for all the available locations). For instance, for a neuron with a preferred word ABC, we collected its activity as a function of the location at which the input word ABC was presented. Then, we selected the more similar location vector using the Euclidean Distance as similarity index. This procedure revealed the number of locations for which the neuron activity was highly similar, that is, the number of neuron's preferred locations. A single preferred location indexes location-specific word coding, whereas 5 preferred locations (i.e., equal preference across locations) indexes location-invariant word coding. Figure 4 shows the distribution of neurons as a function of the number of preferred locations and the word selectivity index (β coefficient of the LD predictor). Notably, location invariance emerged only in the deepest layer (30.83% of HL3 neurons), where word recognition was also near-perfect.

### Bigram coding

To assess whether tuning to bigrams had emerged in the network, we presented all possible sub-word units (i.e., letters and bigrams) to the network and recorded the activation of each neuron within each layer. Letters were presented at all the 7 possible locations, whereas bigrams were divided between contiguous (e.g., AB for ABC) and non-contiguous (e.g., A_C for ABC) and were presented at the 6 and 5 possible locations, respectively. The activity of each neuron across sub-word units was normalized, so that it had a maximum of 1 for its preferred stimulus. We then determined the preferred bigram for each neuron, choosing the bigram that maximized its activity, and performed three diagnostic tests. First, we assessed whether the neuron's responses to both constituent letters were smaller than the response to the bigram by at least 10% (i.e., the neuron's response to AB should be larger than the response to A and to B presented in isolation). Note that we chose a lenient criterion because assuming additivity of the response to the constituent letters (i.e., response to AB as sum of the responses to A and B) is unwarranted for non-linear neurons. Second, we assessed whether it was maximally active for all the words containing the preferred bigram, in order to exclude that the neuron was tuned to specific words. Finally, the candidate bigram neuron was assessed for its sensitivity to letter order. Indeed, a neuron might respond to the co-occurrence of two letters (e.g., A and B), but to be qualified as bigram detector, its response to the transposed letter pair should be smaller (i.e., response should be stronger for AB than for BA). Thus, we computed an index of order sensitivity as the difference between the response to the preferred bigram and to its transposed version presented at the same location. Values close to zero would index lack of order sensitivity, whereas values close to one would show that the neuron does not respond at all to the bigram with the opposite letter order. A neuron passing the first two tests and showing high sensitivity to order would be classified as bigram detector, thereby providing evidence that sensitivity to bigrams has emerged as intermediate coding strategy in the network. This analysis showed that there were no neurons, across the three layers, that could be classified as bigram detectors—indeed, no neuron passed the first two tests.

### Analysis on activation patterns

**Figure 5:**
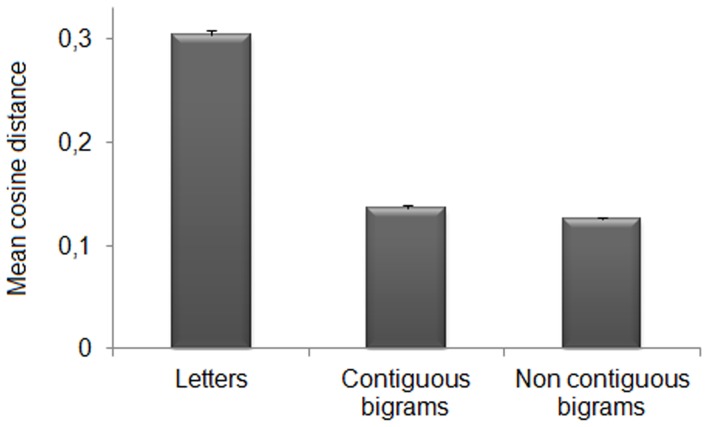
**Pattern analysis on HL3.** Mean cosine distance between internal representations for each word and sub-word units (i.e., letters, contiguous bigrams, and non-contiguous bigrams). Note that smaller values index higher similarity between activation patterns. The sub-word unit showing the highest similarity to the corresponding word was the non-contiguous bigram, that is the combination of exterior letters (word edges).

The contribution of sub-word orthographic units to the representation of words can also be assessed at the level of distributed representations over the hidden neurons of the deepest layer (where word recognition is near-perfect). We therefore, analyzed the similarity between activation patterns produced by training words and those produced by the different types of sub-word units. This analysis was performed on the same network selected for the single neuron analysis (i.e., the first network trained). More specifically, we presented letters and bigrams to the trained network and recorded the pattern of activation of the deepest layer (HL3). Letters and bigrams were presented at all the possible input locations; bigrams were divided between contiguous (e.g., AB and BC for ABC) and non-contiguous (e.g., A_C for ABC). Moreover, letters and bigrams (both contiguous and non-contiguous) could be constituent (e.g., A, B, C, AC, etc. for ABC) or non-constituent (e.g., D, E, F, DE, etc. for ABC). We computed the cosine distance^2^ between the activation pattern produced by each word presented at a randomly selected location and those produced by open bigrams and letters. Note that after fixing the position of the training word, letters and bigrams were presented in the corresponding locations within the word. We then performed a repeated measure analysis of variance on the mean cosine distance, with Unit (3 levels: letters, contiguous bigrams, and non-contiguous bigrams) and Type (2 levels: constituent vs. non-constituent) as factors. Results (see Figure 5) showed significant main effects of Unit, *F*_(2, 238)_ = 78.09, *p* < 0.0001, η^2^_*p*_ = 0.4, and Type, *F*_(1, 119)_ = 23271.38, *p* < 0.0001, η^2^_*p*_ = 0.99. The interaction was also significant, *F*_(1.92, 228.62)_ = 395.31, *p* < 0.0001, η^2^_*p*_ = 0.77 (Huynh-Feldt corrected for sphericity). Paired *t-tests* (Bonferroni corrected) showed that, for each of the sub-word units, there was a higher similarity with constituent than non-constituent units [letters: *t*_(119)_ = −52.39, *p* < 0.0001; contiguous bigrams: *t*_(119)_ = −118.86, *p* < 0.0001; non-contiguous bigrams, *t*_(119)_ = −95.93, *p* < 0.0001]. For constituent units, non-contiguous bigrams had higher similarity to the target words with respect to both single letters [*t*_(119)_ = 39.49, *p* < 0.0001] and contiguous bigrams [*t*_(119)_ = 3.86, *p* < 0.0001]. Contiguous bigrams had higher similarity than letters [*t*_(119)_ = 42.109, *p* < 0.0001]. For non-constituent units, only single letters showed a significant difference from the other sub-word units [contiguous bigrams, *t*_(119)_ = −6.64, *p* < 0.0001; non-contiguous bigrams, *t*_(119)_ = −4.28, *p* < 0.0001].

In summary, the activation pattern of each word was more similar to those of the constituent rather than those of non-constituent sub-word units. Importantly, among constituent units, non-contiguous bigrams (i.e., those formed by the first and last letter) produced an activation pattern that was more similar to that of the corresponding word in comparison to both letters and contiguous bigrams. For constituent letters and contiguous-bigrams we performed a further analysis (i.e., two-tailed paired *t*-tests, Bonferroni corrected), in order to establish whether the position of the constituent stimuli within the word was important. Results revealed no significant differences among positions for both letters (first letter: *M* = 0.31 ± 0.005 s.e.m., second letter: *M* = 0.30 ± 0.005 s.e.m., third letter: *M* = 0.30 ± 0.004 s.e.m.; all *t*s < 2.01) and contiguous bigrams (first bigram: *M* = 0.14 ± 0.003 s.e.m., second bigram: *M* = 0.13 ± 0.002 s.e.m.; *t* = 1.55). We also assessed whether the higher similarity of the non-contiguous bigram pattern to the word pattern with respect to the continuous bigram patterns would persist when the leftmost and rightmost word locations (1 and 5) were excluded from the analysis. The results did not change [non-contiguous vs. contiguous bigrams: *t*_(119)_ = 3.71, *p* < 0.0001].

## Discussion

Models of orthographic coding (e.g., Grainger and van Heuven, 2003; Grainger and Whitney, 2004; Gomez et al., 2008; Davis, 2010) share the assumption that visual word recognition is performed through the processing of constituent letters but differ on how letter position information is coded and whether the mapping between location-specific (eye-centered) letter coding and location-invariant word representations requires the computation of an intermediate orthographic code, such as open bigrams. A prior attempt to tackle these issues through a connectionist approach has led to contrasting results. After training a feedforward neural network with one hidden layer (using error back-propagation) on the mapping between a location-specific letter code and location-invariant localist word representations, Dandurand et al. (2010) showed computational evidence supporting the bigram coding hypothesis. However, subsequent analyses of the hidden layer representations carried out by Hannagan et al. (2011) suggested that in this network model the mapping does not imply the extraction of information about letter combinations but it is based on semi-location-invariant letter representations that are broadly consistent with the overlap model of Gomez et al. (2008).

Our current attempt to use connectionist simulations for cracking the orthographic code is tied to a more general framework suggesting that perceptual invariance can emerge from unsupervised learning in a hierarchical processing architecture that extracts increasingly more complex and abstract features (Hinton, 2007, 2013; Stoianov and Zorzi, 2012; Zorzi et al., 2013), as well as to the hypothesis that visual word recognition recycles the cortical machinery used for visual object recognition (Dehaene and Cohen, 2011; Dehaene et al., 2005). Accordingly, we exploited deep neural networks (Hinton and Salakhutdinov, 2006) to investigate whether location-invariant word recognition might emerge from unsupervised learning of a hierarchical generative model of location-specific letter patterns. Although word-level information (i.e., word identity) was never provided to the network during training, linear decoding from the activity of the deepest hidden layer yielded near-perfect accuracy in location-invariant word recognition. In contrast, decoding accuracy from lower HLs showed a sharp and progressive decrease, with a pattern of errors suggesting that letter position information was not coded in a location-invariant way. Indeed, the majority of the word decoding errors, especially at the second hidden layer, consisted of transposition errors. This finding is consistent with the transposition priming effect, as predicted by both letter-based (e.g., Gomez et al., 2008; Davis, 2010) and open bigram (e.g., Grainger and van Heuven, 2003; Grainger and Whitney, 2004) models of orthographic coding.

We then carried out a series of analyses to investigate the nature of the orthographic representations emerged at the different HLs. Analysis on single neurons showed that only the deepest layer of the deep network contained neurons that were both word selective and location-invariant. Interestingly, some word-selective neurons found at the second hidden layer were tuned to specific word locations. These results are in line with those provided by the decoding analysis and confirm that linear decoding of hidden layer activity is an helpful method for investigating the internal representations emerged in a deep network model. Notably, the single neuron analysis showed that bigrams did not emerge as unit of representation in the network. This finding fits well the results of Hannagan et al. (2011) in their re-analysis of the Dandurand et al. (2010) model and it is broadly consistent with letter-based models of orthographic coding (Gomez et al., 2008; Davis, 2010). It is worth noting that learning a generative model is equivalent to discovering efficient ways of coding the input data (Ghahramani et al., 1999); this suggests that the information carried by bigrams is not necessary for efficient orthographic coding, at least in the context of the highly constrained training set employed in the present study (see further discussion below).

In a final set of analyses, we recorded the activation patterns over the deepest hidden layer produced by each word and compared them to those produced by letters and bigrams. This analysis provides a measure of similarity between internal representations that can be readily interpreted in terms of priming effect. Not surprisingly, constituent letters and bigrams (including non-contiguous bigrams) had an advantage over non-constituent ones. Moreover, constituent bigrams had an advantage over constituent letters, which is also expected due the increasing orthographic overlap (i.e., two letters vs. one letter). Interestingly, we also found a significant greater similarity for non-contiguous bigrams (i.e., those formed by the first and last letter) over contiguous bigrams. The advantage for non-contiguous bigrams persisted when the extreme word locations (1 and 5) were excluded from the analysis. The superiority of non-contiguous bigrams with respect to the other constituent stimuli might be interpreted as an index of the edge effect (Fischer-Baum et al., 2011), that is the superiority of the first and last letters for coding words as a sequence of ordered letters, observed using the illusory word paradigm. Fischer-Baum and colleagues argued that the edge effect supports an orthographic coding scheme in which the beginning and the end letters of a word act as anchoring points. Though several models assume that the exterior letters have special status in orthographic coding (e.g., Gomez et al., 2008; Davis, 2010), our model shows that this aspect is an emergent property that does not require additional mechanisms or specific parameters.

In conclusion, our study shows that location-invariant visual word recognition can emerge from unsupervised learning in a neural network with a deep (hierarchical) architecture. Our deep network model extracted increasingly more complex and abstract orthographic features across layers. Moreover, our analyses show that the emergent orthographic code is not based on bigrams and it assigns special status to the exterior letters (word edges). Although restricting our simulations to an artificial dataset of 3-letter strings is indeed an important limit of the current study, this allowed us to investigate orthographic coding in a simplified and tightly controlled way. Future extensions of this work will therefore focus on scaling-up the training dataset and on testing the model on a corpus of real words. For example, it cannot be excluded that the distributional statistics of letters in real words, whereby some letter combinations have higher frequency than others, might lead to the emergence of sub-word units like bigrams (see Dandurand et al., 2011, for analyses of English, French and Spanish word corpora). Nevertheless, we believe that our preliminary findings pave the way for a better understanding of how orthographic representations can emerge through unsupervised learning within a sound probabilistic framework.

### Conflict of interest statement

The authors declare that the research was conducted in the absence of any commercial or financial relationships that could be construed as a potential conflict of interest.

## Footnotes

^1^Note that using a more compact representation with only six letter units (for letters A–F) at each slot does not change the results presented here. Conversely, the use of a full set of letter units allows to readily extend the model's training set.

^2^Cosine distance between patterns X and Y is calculated as 1-cosine (X, Y).
